# Development of Phenotypic and Transcriptional Biomarkers to Evaluate Relative Activity of Potentially Estrogenic Chemicals in Ovariectomized Mice

**DOI:** 10.1289/ehp.1307935

**Published:** 2015-01-09

**Authors:** Sylvia C. Hewitt, Wipawee Winuthayanon, Brianna Pockette, Robnet T. Kerns, Julie F. Foley, Norris Flagler, Elizabeth Ney, Apichart Suksamrarn, Pawinee Piyachaturawat, Pierre R. Bushel, Kenneth S. Korach

**Affiliations:** 1 Receptor Biology, Reproductive and Developmental Biology Laboratory; 2 Microarray and Genome Informatics Group, National Institute of Environmental Health Sciences (NIEHS), National Institutes of Health (NIH), Department of Health and Human Services (DHHS), Research Triangle Park, North Carolina, USA; 3 Kelly Government Solutions Inc., Morrisville, North Carolina, USA; 4 Cellular and Molecular Pathology Branch, NIEHS, NIH, DHHS, Research Triangle Park, North Carolina, USA; 5 Department of Chemistry, Faculty of Science, Ramkhamhaeng University, Bangkok, Thailand; 6 Department of Physiology, Faculty of Science, Mahidol University, Bangkok, Thailand; 7 Biostatistics Branch, NIEHS, NIH, DHHS, Research Triangle Park, North Carolina, USA

## Abstract

**Background:**

Concerns regarding potential endocrine-disrupting chemicals (EDCs) have led to a need for methods to evaluate candidate estrogenic chemicals. Our previous evaluations of two such EDCs revealed a response similar to that of estradiol (E_2_) at 2 hr, but a less robust response at 24 hr, similar to the short-acting estrogen estriol (E_3_).

**Objectives:**

Microarray analysis using tools to recognize patterns of response have been utilized in the cancer field to develop biomarker panels of transcripts for diagnosis and selection of treatments most likely to be effective. Biological effects elicited by long- versus short-acting estrogens greatly affect the risks associated with exposures; therefore, we sought to develop tools to predict the ability of chemicals to maintain estrogenic responses.

**Methods:**

We used biological end points in uterine tissue and a signature pattern–recognizing tool that identified coexpressed transcripts to develop and test a panel of transcripts in order to classify potentially estrogenic compounds using an *in vivo* system. The end points used are relevant to uterine tissue, but the resulting classification of the compounds is important for other sensitive tissues and species.

**Results:**

We evaluated biological and transcriptional end points with proven short- and long-acting estrogens and verified the use of our approach using a phytoestrogen. With our model, we were able to classify the diarylheptanoid D3 as a short-acting estrogen.

**Conclusions:**

We have developed a panel of transcripts as biomarkers which, together with biological end points, might be used to screen and evaluate potentially estrogenic chemicals and infer mode of activity.

**Citation:**

Hewitt SC, Winuthayanon W, Pockette B, Kerns RT, Foley JF, Flagler N, Ney E, Suksamrarn A, Piyachaturawat P, Bushel PR, Korach KS. 2015. Development of phenotypic and transcriptional biomarkers to evaluate relative activity of potentially estrogenic chemicals in ovariectomized mice. Environ Health Perspect 123:344–352; http://dx.doi.org/10.1289/ehp.1307935

## Introduction

The ovariectomized mouse uterus exhibits rapid biochemical and biological responses to estrogens that have been extensively studied and characterized (Katzenellenbogen et al. 1979). Using this mouse model, we previously evaluated transcript responses by microarray and identified several thousand genes with transcripts that are increased or decreased following estrogen treatment (Hewitt et al. 2003). Natural and synthetic estrogenic substances are known to exhibit characteristic degrees of activity. For example, long-acting estrogens, such as estradiol (E_2_) and diethylstilbestrol (DES), elicit biological responses early (within 1–2 hr) and persist, resulting in later responses (24–72 hr) that lead to maximal uterine growth. In contrast, short-acting estrogens, such as estriol (E_3_), match long-acting estrogens in eliciting the early responses, but because of their pharmacokinetics they do not persist, and thus *in vivo* responses characteristic of later end points are blunted (Clark and Markaverich 1984; Katzenellenbogen 1984). However, E_3_ is able to match E_2_ in *in vitro* systems: For example, E_3_ is able to stimulate MCF-7 cell growth because culture conditions lack E_3_ metabolic clearing (Katzenellenbogen 1984). The xenoestrogens bisphenol A (BPA) and 2,2-bis(*p*-hydroxyphenyl)-1,1,1-trichloroethane (HPTE) elicited uterine transcription patterns similar to those of the proven short-acting estrogen E_3_ (Hewitt and Korach 2011; Klotz et al. 2000). Responses were highly correlated to those of E_2_ at 2 hr but were attenuated and less correlated to E_2_ at 24 hr (Hewitt and Korach 2011). We confirmed the short-acting estrogen-like activities of BPA and HPTE by observing uterine biological responses that were characteristic of short-acting estrogens. These end points included uterine weight increases that were attenuated after 72 hr (3 days) and decreased induction of epithelial cell DNA synthesis after 24 hr (Hewitt and Korach 2011).

Recognizing not only the potential estrogenic activity of endocrine-disrupting chemicals (EDCs) but also determining whether they have short-acting or sustained actions is important for evaluation of risks to humans and wildlife. Because of their more sustained activity (Hewitt and Korach 2011; Safe et al. 2001), long-acting estrogens are more likely to affect estrogen-sensitive tissues and thus might be considered of greater risk in terms of their impacts on exposed populations (Safe et al. 2001). In contrast, individuals who lack endogenous long-acting estrogens, including prepubertal girls and postmenopausal women, might also exhibit susceptibility to short-acting estrogens. It is therefore important to develop tools predicting estrogenicity and also potential to elicit sustained responses. In the study of cancer, biomarker panels of transcripts have been developed as tools for diagnosis and for selection of treatments most likely to be effective (Gormley et al. 2007). In the present study, we aimed to use the well-characterized ovariectomized mouse uterus model to generate a screen that incorporates phenotypic estrogen-response end points and transcriptional biomarkers that distinguish estrogenic characteristics. We hope our approach will allow more detailed assessment of substances than do *in vitro* high throughput screens but without undertaking extensive animal studies or comprehensive genomic analyses. We developed panels of transcripts for use as biomarkers and phenotypic uterine responses to assess the activity of candidate compounds in terms of estrogenic activity and classification as short- versus long-acting estrogen. We analyzed uterine microarray data sets using the EPIG tool (Extracting Patterns and Identifying co-expressed Genes; Chou et al. 2007), and we identified patterns characteristic of short-acting (only 2 hr) or long-acting (both 2 hr and 24 hr) estrogen response. To increase the power of validation of potential biomarker transcripts, we used biological end points including uterine weight, proliferation and/or apoptosis of epithelial cells, increase in luminal epithelial cell height, and induction of apoptosis inhibitor, thus providing phenotypic anchoring to support transcriptional responses. We identified panels of 50 genes each at 2 or 24 hr using a combined analysis of variance (ANOVA) and principal component analysis (PCA) approach, and we validated the panels by establishing activity of a candidate compound. The use of these biomarkers and response end points (summarized in Table 1) will allow evaluation of potential estrogenic mechanisms for chemicals of environmental concern in a biological system. The diarylheptanoid D3 [(3R)-1,7-diphenyl-(4E,6E)-4,6-heptadien-3-ol], a natural phytoestrogen isolated from an indigenous plant [*Curcuma comosa* Roxb. (Zingiberaceae family)] used by postmenopausal women in Thailand, was previously characterized to exhibit estrogen-like activity in the uterus with a short-acting biological action compared with E_2_ (Winuthayanon et al. 2009, 2013). Therefore, we used D3 as a candidate to test our panel.

**Table 1:** Scheme for screening potential estrogenic substances using phenotypic and transcriptional end points.

Type of compound	2-hrRNA panel^*a*^	24-hrEdU^*a*^	24-hrweight^*a*^	72-hrweight^*b*^	24-hrRNA panel^*a*^	72-hrTUNEL^*b*^	72-hr*Birc1a*^*b*^	72-hr*Ltf*^*b*^	72-hrEpi cell height^*b*^
Non-E	–	–	–	–	–	–	–	–	–
Short-acting E	+	+	–/+	–/+	–/+	+	–/+	–/+	–/+
Long-acting E	+	++	+	++	++	–	++	++	++
Abbreviations: –, no response; –/+, some or inconsistent response; +, moderate response; ++, strong response; E, estrogen; Epi, epithelial. ^***a***^Time after a single injection. ^***b***^Seventy-two hours after the first of three daily injections.									

## Materials and Methods

*Uterine bioassays*. Mice were housed three to five per cage in static microisolator solid-bottom cages (Super Mouse 750™ cages; Lab Products) on autoclaved hardwood bedding (Sani-chips; PJ Murphy Forest Products Corp.) and maintained on a 12:12-hr light:dark cycle at 22 ± 0.5°C and relative humidity of 40–60%. Mice were provided with autoclaved rodent diet (NIH31; Zeigler Brothers) and reverse-osmosis–treated deionized water *ad libitum*. All procedures were reviewed and approved by the National Institute of Environmental Health Sciences Animal Care and Use Committee. All animals were housed, cared for, and used in compliance with the *Guide for the Care and Use of Laboratory Animals* (National Research Council 2011) in a program accredited by the Association for Assessment and Accreditation of Laboratory Animal Care. Animals were treated humanely and with regard for alleviation of suffering according to NIEHS Animal Care and Use Committee Guidelines and in compliance with a NIEHS-approved animal protocol. Ovariectomized adult C57BL/6 mice (*n* = 211; 8–10 weeks of age) were obtained from Charles River Laboratories and shipped to the NIEHS facility. Animals were then housed five per cage for 10–14 days after surgery to clear circulating ovarian hormones before treatment began. Mice were assigned to treatment groups by cage mates, or in cases where fewer than five per goup were used, mice were divided by randomly removing some mice and combining them in a new cage.

For 2- and 24-hr samples [24 hr for uterine weight, 5-ethynyl-2´-deoxyuridine (EdU) incorporation, and reverse-transcription polymerase chain reaction (RT-PCR); 2 hr for RT-PCR], mice were injected with vehicle [100 μL sterile saline (0.85% NaCl) plus 0.1% ethanol (*n =* 5)]; E_2_ (Steraloids; 2 hr, *n =* 4; 24 hr, *n* = 5), E_3_ (Steraloids; 2 hr, *n =* 4; 24 hr, *n =*5), or DES (Sigma; 24 hr only; *n* = 3), 1-mg/mL 100% ethanol (EtOH) stock solution diluted in saline to a concentration of 250 ng/100 μL; or 4,4´,4´´-(4-propyl-[1H]-pyrazole-1,3,5-triyl)trisphenol (PPT; Tocris Bioscience; 24 hr only, *n* = 3) 50-mg/mL 100% EtOH stock solution diluted to a concentration of 250 μg in 100 μL saline + 10% Cremaphor EL (Sigma). Treatments were administered by intraperitoneal injection.

The diarylheptanoid D3 was isolated and administered as previously described (Suksamrarn et al. 2008; Winuthayanon et al. 2009, 2013). For the D3 experiments, mice were treated with sesame oil (Sigma) plus 10% EtOH (vehicle; *n =* 5); D3 [250-mg/mL 100% EtOH stock solution diluted to a concentration of 2.5 mg/100 μL sesame oil (2 hr, *n =* 4; 24 hr, *n =* 7)]; or E_2_ [1-mg/mL 100% EtOH stock solution diluted in sesame oil with added ethanol to a concentration of 250 ng E_2_ in 100 μL sesame oil plus 10% ethanol (2 hr, *n =* 4; 24 hr, *n =* 7)]. Treatments were administered by subcutaneous injection. Doses of all compounds were selected based on the minimum dose required to induce optimal uterine responses. For E_2_, DES, E_3_, and D3, see Supplemental Material, Figure S1. The PPT dose is based on responses reported by Sinkevicius et al. (2008).

For 2-hr experiments, treatments were given without regard to time of day. For all 24-hr experiments, all injections and collections were done in the morning. EdU (2 mg in 100 μL phosphate-buffered saline; Life Technologies) was injected intraperitoneally 22 hr after hormone injections, and uterine tissue was collected 2 hr later (24 hr after hormone treatment). For 72-hr (3-day) end points (vehicle, *n =* 7; E_2_, *n =* 7; E_3_, *n =* 4; DES, *n =* 4; PPT, *n =* 4; and for D3 experiments (vehicle, *n =* 8; E_2_, *n =* 8; D3, *n =* 8), the same concentrations were used as for 24-hr end points, but all were diluted in sesame oil (Sigma) and injected subcutaneously each day for 3 days. Injections for 72-hr experiments were initiated without regard for time of day, but were always administered 24 hr apart, and uterine tissues were collected on the fourth day. For both 24-hr and 72-hr experiments, uterine tissue was weighed, with a portion of one horn fixed in 10% formalin (Fisher) and the remainder snap frozen in liquid nitrogen. For 2-hr experiments, uterine tissue was snap frozen in liquid nitrogen. Frozen uterine tissue was processed for RNA analysis by real-time RT-PCR as previously described (Hewitt et al. 2003). We used at least three animals per group based on previous studies indicating that significant changes in end points are likely to be detected (Hewitt and Korach 2011).

Fixed tissue was processed either to detect incorporated EdU using the ClickIT Kit (Life Technologies) or to indicate terminal deoxynucleotidyl transferase dUTP nick end labeling (TUNEL)-positive apoptotic cells using the ApopTag Plus Peroxidase In Situ Apoptosis Kit (catalog no. S7101; Millipore), both methods described previously by Winuthayanon et al. (2010). Image analysis to evaluate cell proliferation and apoptosis was completed on transverse sections of EdU and TUNEL slides scanned with an Aperio Scanscope XT Digital Slide Scanner (Leica Biosystems) and viewed using an Aperio® ImageScope v. 12.0.1.5027 (Aperio Technologies Inc.). We determined percent positive EdU staining by counting total and EdU Alexafluor 488–positive uterine luminal cells within the total uterine luminal area. The total uterine luminal area was selected by outlining the basement membrane of the uterine luminal epithelium. The percentage of luminal cells that were TUNEL positive was determined by counting the number of TUNEL-positive cells per total epithelial cells in six regions of the uterine lumen (300–800 cells per slide). Luminal epithelial cell height (LEH) was measured in five areas per transverse section using the Ruler tool in ImageScope™. Data files were exported to Excel™ (v.14.3.9; Microsoft) and compiled into a single worksheet.

*Microarray analyses*. We used data from previous microarray studies for pattern analysis [Gene Expression Omnibus (GEO; http://www.ncbi.nlm.nih.gov/geo/) accession numbers GSE24525, GSE18168, GSE23072, GSE23241, and GSE61921S; see summary in Supplemental Material, Tables S1 and S2]. Data from the GEO database included vehicle controls (11 samples), E_2_ (13 samples), E_3_ (6 samples), DES (4 samples), and PPT (4 samples) for 2 or 24 hr. All were generated using Agilent mouse 4 × 44K array chips (Agilent Technologies), and data were acquired and analyzed as described previously (Hewitt and Korach 2011; Hewitt et al. 2009, 2010, 2012).

**Figure 1:**
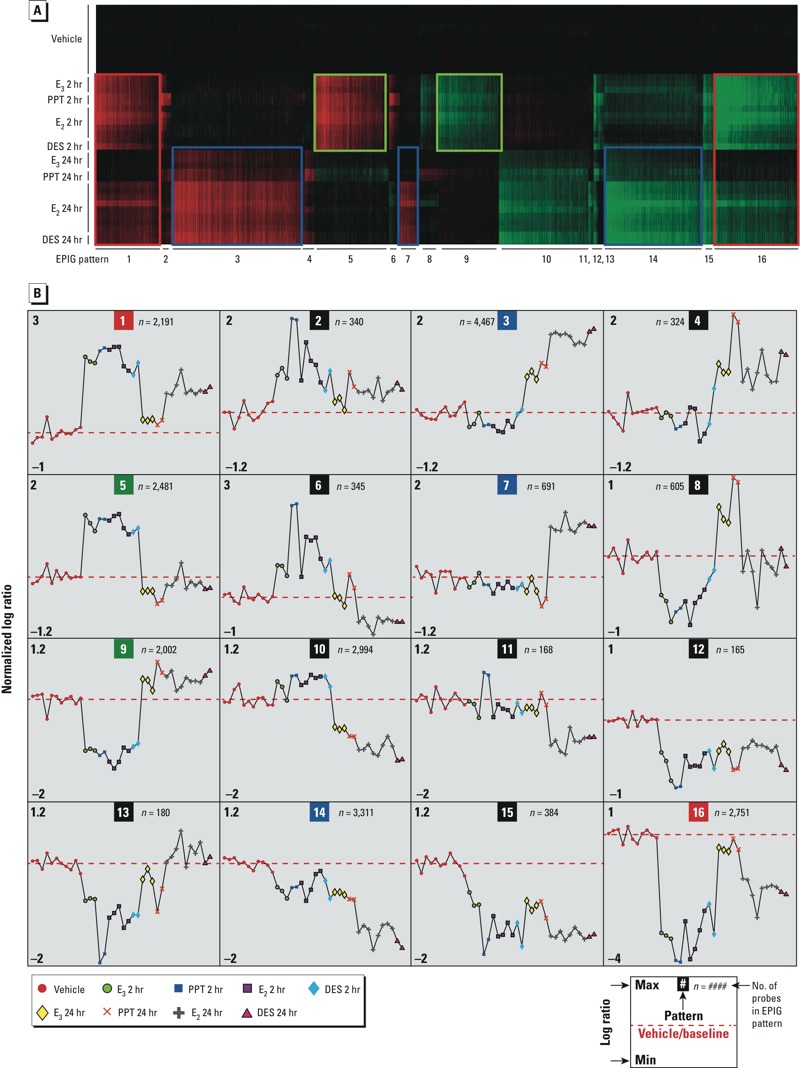
Microarray data sets analyzed by EPIG to extract expression patterns. Mice were treated with either saline vehicle, 24 hr (11 samples); E_3_, 2 hr or 24 hr (3 samples each), PPT, 2 hr or 24 hr (2 samples each), E_2_, 2 hr (5 samples) or 24 hr (8 samples); or DES, 2 hr or 24 hr (2 samples each). (*A*) Microarray data sets with coregulation patterns identified; numbers across the bottom indicate the 16 patterns that were extracted. Green boxes outline patterns 5 and 9; blue boxes outline patterns 3, 7, and 14; and red boxes outline patterns 1 and 16. (*B*) Sixteen patterns extracted by EPIG. The inset in the lower right corner describes information shown in each box. Abbreviations: Max, maximum; Min, minimum.

*EPIG analysis and derivation of biomarker panels*. Batch-corrected microarray data sets (see Supplemental Material, “EPIG Analysis” and Figure S4) were analyzed using the EPIG tool (Chou et al. 2007) resulting in 16 patterns (Figure 1A,B). Probes that could distinguish estrogenic from nonestrogenic treatments (2 hr; 50 probes) or long-acting from short-acting estrogens (24 hr; 50 probes) were derived as described in Supplemental Material, “Deriving biomarker panels.” Hierarchical clustering of the expression of the 2-hr or 24-hr probes with estrogens validated their selection (see Supplemental Material, Figure S2 and Tables S3 and S4).

*RT-PCR and NanoString screen*. We selected probes to test the 2-hr and 24-hr biosets by RT-PCR by first combining the 50 probe panels validated above (see Supplemental Material, Figure S2) with 26 additional 2-hr probes or 12 additional 24-hr probes covering RT-PCR primer sets for previously studied estrogen-responsive transcripts, and these probes were analyzed using Ingenuity Pathway Analysis (http://www.ingenuity.com/products/ipa). Probes that duplicated gene names and genes that represented the same biological function were consolidated. Genes for RT-PCR validation were then selected using a random number generator. For NanoString analysis, 129 biomarker probes plus 9 housekeeping probes were submitted to NanoString Technologies for CodeSet design, and RNA samples were shipped to NanoString for processing and analysis. Briefly, 100 ng of each RNA sample was added to the CodeSet in hybridization buffer and incubated at 65°C for 16 hr. The CodeSet consists of Reporter and Capture probes that hybridize to the target sequences of interest, forming a tripartite complex. Assays were purified using the Prep Station software (High Sensitivity Protocol; NanoString), and data were collected using the GEN2 Digital Analyzer (NanoString), and raw counts were provided. Raw counts were then were normalized using nSolver software (NanoString) and exported into Partek Genomics Suite 6.6 (Partek Inc.) for clustering. Normalized values are presented in Supplemental Material, Supplemental XLSX, Table S5.

*Statistical analysis for biological end points*. Data for real-time RT-PCR as well as quantification of EdU and TUNEL-positive cells and uterine LEH are presented as mean ± SE. GraphPad Prism 6.0 for Windows (http://www.graphpad.com/scientific-software/prism/) was used for statistical analysis. Differences were considered significant when *p* < 0.05, using ANOVA with Tukey multiple comparisons post hoc, unless otherwise indicated.

## Results

**Figure 2:**
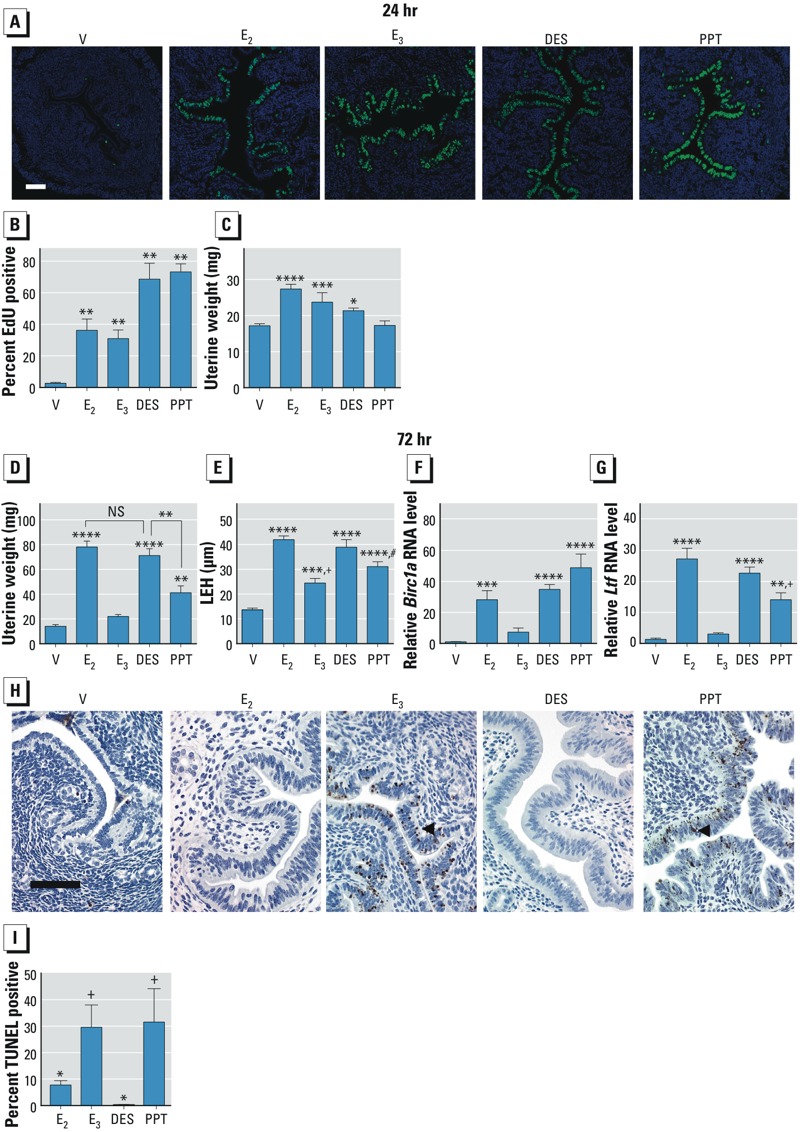
Phenotypic end points of estrogenic response in the uterus of mice treated with saline vehicle (V), E_2_, E_3_, DES, or PPT for 24 (*A–C*) or 72 hr (*D–I*). (*A*) Representative photomicrographs showing cell proliferation 24 hr after treatment, indicated by incorporation of EdU (green); blue indicates Hoescht staining of DNA. Uterine epithelial cells show active DNA synthesis, and basal EdU incorporation is present in vehicle-treated mice; bar = 0.1 mm. (*B*) Percentage of EdU-positive cells in uterine tissue 24 hr after injection (mean ± SE; *n* = 5–9 mice/group). **p < 0.01 compared with V by one-way ANOVA with multiple comparisons and uncorrected Fisher’s least significant difference (LSD). (*C*) Uterine weight 24 hr after injection (mean ± SE; *n* = 3–5 mice/group). *p < 0.05, ***p < 0.001, and ****p < 0.0001, compared with V by one-way ANOVA, with multiple comparisons, with Tukey’s multiple test correction. (*D*) Uterine weight in the 72 hr (3 day) bioassay, with uteri collected 24 hr after the last of three daily injections (mean ± SE; *n* = 4–7 mice/group). **p < 0.001 for PPT compared with DES or E_2_; and ****p < 0.0001 compared with V, by one-way ANOVA, with multiple comparisons, with Tukey’s multiple test correction. (*E*) Luminal epithelial cell height (LEH) in the 72‑hr bioassay (mean ± SE; *n* = 4–7 mice/group). ***p <0.001, and ****p < 0.0001 compared with V; ^+^*p* < 0.0001 compared with E_2_ or DES; and ^#^*p* < 0.001 compared with E_2_ by one-way ANOVA, with multiple comparisons, with Tukey’s multiple test correction. (*F*) Level of RNA for the apoptosis inhibitor *Birc1a *measured by RT‑PCR in uteri from the 72‑hr bioassay (mean ± SE; *n* = 4–7 mice/group).*****p < 0.001, and ****p < 0.0001 compared with V by one-way ANOVA with multiple comparisons and uncorrected Fisher’s LSD. (*G*) *Ltf* transcript measured by RT-PCR in uteri from the 72‑hr bioassay (mean ± SE; *n* = 4–7 mice/group). **p < 0.01, ***p < 0.001, and **** *p* < 0.0001 compared with V; and ^+^*p* < 0.01 for PPT compared with E_2_ by one-way ANOVA, with multiple comparisons, with Tukey’s multiple test correction. (*H*) Representative photomicrographs showing apoptotic cells in uteri from the 72‑hr bioassay as indicated by the TUNEL assay; bar = 0.1 mm. Each arrowhead points to a TUNEL-positive cell. (*I*) Percent TUNEL-positive luminal epithelial cells (mean ± SE; *n* = 4–10 mice/group.

*Biological end points*. We compared proliferation of uterine epithelial cells—a hallmark of estrogenic response—in ovariectomized mice treated with E_2_, E_3_, DES (a synthetic estrogen), or PPT [a synthetic estrogen receptor α (ERα)–selective agonist]. Treatment with all of the estrogens resulted in an increase in epithelial cells in S phase, as reflected by incorporation of the thymidine triphosphate analog EdU (Figure 2A,B). At the 24-hr time point, uterine wet weights were increased by E_2_, E_3_, or DES (*p* < 0.05), but not significantly by PPT (Figure 2C). However, after 3 days of treatment, uterine weight was not significantly increased by E_3_; however, E_2_ or DES treatment led to a robust uterine weight increase (*p* < 0.0001), whereas PPT caused an increase (*p* < 0.001) that was significantly lower than the increase induced by E_2_ or DES (*p* < 0.001) (Figure 2D). In the 72-hr bioassay, E_2_, E_3_, DES, and PPT treatment resulted in increased epithelial thickness and cell height (*p* < 0.001; Figure 2E); however, PPT and E_3_ increased the epithelial thickness significantly less than did E_2_ (*p* < 0.001 vs. E_2_; Figure 2E). Three days of E_3_ treatment did not induce transcripts of either the apoptosis inhibitor *Birc1a* (baculoviral IAP repeat-containing protein 1a) or the epithelial cell secretory protein *Ltf* (lactoferrin), whereas E_2_, DES, and PPT induced both of these (Figure 2F,G). In addition, E_3_ and PPT exhibited significantly more TUNEL-positive epithelial cells than E_2_ or DES (*p* < 0.05; Figure 2H,I), indicating increased apoptosis.

*Bioinformatic analysis to develop transcript panels*. Toward our goal of developing panels of biomarker transcripts, which, in combination with these biological end points, might be used to screen for potential estrogenic activity of candidate chemicals, we used the EPIG tool (Chou et al. 2007) to identify patterns of gene expression in our microarray data sets. Because our data sets did not include sufficient numbers of test compounds for derivation of training and validation nor did they include test data sets to appropriately perform a prediction, we utilized a phenotypic anchoring approach. We correlated gene expression according to the samples defined by the strength of estrogen response. The statistical framework of this correlation has been well described (Chou et al. 2007) and has statistical properties that are similar to an ANOVA model (sample variances), *t*-test (group comparisons), and signal processing (signal-to-noise thresholding). However, our approach also leverages the biological responses of the samples within a group, and correlation of gene expression among the groups, to extract patterns of genes that have a low probability of being detected by chance. Hence, the statistical rigor in our methodology supports the selection of these biomarker transcripts combined with these biological end points as a potential screen for estrogenic activity of candidate chemicals (Table 1). Figure 1A shows patterns of transcript profiles in uterine tissue from mice treated with vehicle, PPT, E_3_, DES, or E_2_ for 2 or 24 hr. Each plot in Figure 1B represents one of the 16 response patterns extracted by EPIG. For example, the estrogenic compounds (E_3_, PPT, E_2_, and DES) increased the levels of the 2,481 probes in pattern 5 relative to vehicle after 2 hr, but the level of these probes returned to baseline by 24 hr. Similarly, the estrogenic compounds decreased the levels of the 2,002 probes in pattern 9 after 2 hr. The probes in these patterns could thus be used to examine whether a substance has estrogenic activity by evaluating the levels of these transcripts 2 hr after treatment. After 24 hr of treatment, E_2_ or DES more robustly increased the 4,467 probes in pattern 3 relative to vehicle than did PPT or E_3_. Only E_2_ or DES, but not PPT or E_3_, increased the 691 probes in pattern 7. After 24 hr of treatment, both E_2_ and DES repressed the 3,311 probes in pattern 14 relative to vehicle, whereas PPT and E_3_ were less effective. Therefore, long-acting versus short-acting estrogens could be distinguished by evaluating the levels of transcripts from patterns 3, 7, or 14 at 24 hr after treatment. After 2 hr of treatment, all of the compounds increased the levels of the 2,191 probes in pattern 1. However, after 24 hr, only DES and E_2_ maintained the increased levels of the probes, whereas the levels were lower for the short-acting estrogens. After 2 hr of treatment, all of the compounds decreased the levels of the 2,751 probes in pattern 16; however, after 24 hr, only DES and E_2_ maintained the repressed levels of the probes, but the short-acting estrogens returned to the vehicle level. Therefore, probes in patterns 1 and 16 could demonstrate estrogenic response at 2 hr and distinguish short-acting from long-acting estrogens at 24 hr.

Two panels, each comprising 50 probes, were selected using a combined ANOVA and PCA approach, detailed in the Supplemental Material, “Deriving biomarker panels.” The first panel could be used to indicate potentially estrogenic substances based on their transcriptional response after 2 hr compared with vehicle-treated samples. The second panel could be used to indicate whether the estrogenic substance was short acting or long acting, based on the response at 24 hr. Our previous work indicated that BPA and HPTE are short-acting estrogens (Hewitt and Korach 2011). We used our previous microarray data sets (E_2_, E_3_, DES, BPA, HPTE, and PPT) to examine the expression of the probes in the biomarker panels. Supplemental Material, Figure S2A shows the level of the 50 probes 2 hr after treatment with E_2_, E_3_, DES, BPA, HPTE, or PPT relative to vehicle (see Supplemental Material, Supplemental XLSX, Table S6); all of the compounds showed responses distinct from vehicle at this time point. Supplemental Material, Figure S2B (see also Supplemental Material, Supplemental XLSX, Table S7) shows the 50 probes selected to distinguish long-acting estrogens (E_2_ and DES) from short-acting estrogens (E_3_, BPA, and HPTE). PPT was not included in the short- versus long-acting 24-hr panel analysis, because it is an ERα selective agonist but has not been classified as short- or long-acting. The biological data (Figure 2) indicated that PPT exhibited a response between long- and short-acting estrogens.

**Figure 3:**
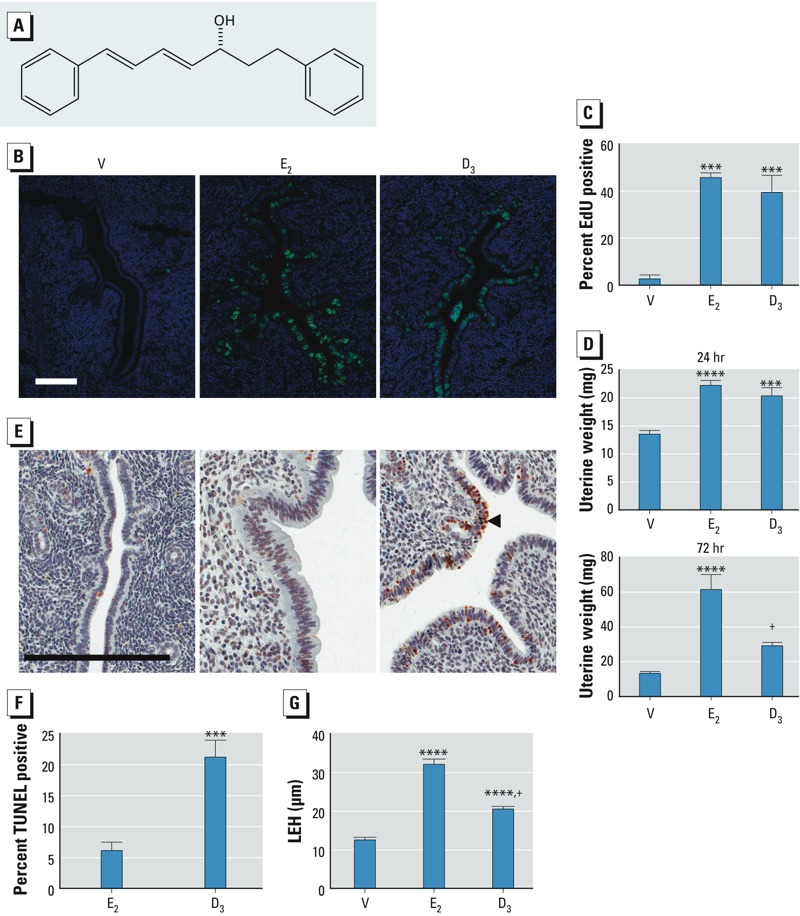
Effects of saline vehicle (V), E_2_, and D3 after treatment for 24‑hr (*B–D*) or 72 hr (*E–G*). (*A*) Structure of D3. (*B*) Representative photomicrographs showing EdU incorporation in uterine epithelial cells 24 hr after treatment bar = 0.1 mm. (*C*) Percentage of EdU-positive cells in uterine epithelial cells 24 hr after treatment (mean ± SE; *n* = 5–7 mice/group). ***p < 0.001 compared with V by one-way ANOVA with uncorrected Fisher’s least significant difference (LSD). (*D*) Uterine weight (mean ± SE) at 24 hr after treatment (top; *n* = 9 mice/group) or 72 hr (24 hr after the last of three daily injections; bottom; *n *= 5 mice/group). ***p < 0.001, and ****p < 0.0001 compared with V, and ^+^*p* < 0.01 compared with D3 by one-way ANOVA with uncorrected Fisher’s LSD. (*E*) Representative photomicrographs showing apoptotic cells in uteri collected at 72 hr (24 hr after the last of three daily injections) as determined by the TUNEL assay (bar = 0.2 mm; the arrowhead points to a TUNEL-positive cell. (*F*) Percentage of TUNEL-positive cells in uteri collected at 72 hr (24 hr after the last of three daily injections; mean ± SE; *n* = 8 mice/group). ***p < 0.001 by unpaired *t*-test. (*G*) LEH in uteri collected at 72 hr (24 hr after the last of three daily injections; mean ± SE; *n* = 8 mice/group). ****p < 0.0001 compared with V, and ^+^*p* < 0.001 compared with E_2_ by one-way ANOVA with Tukey’s multiple comparison test.

**Figure 4:**
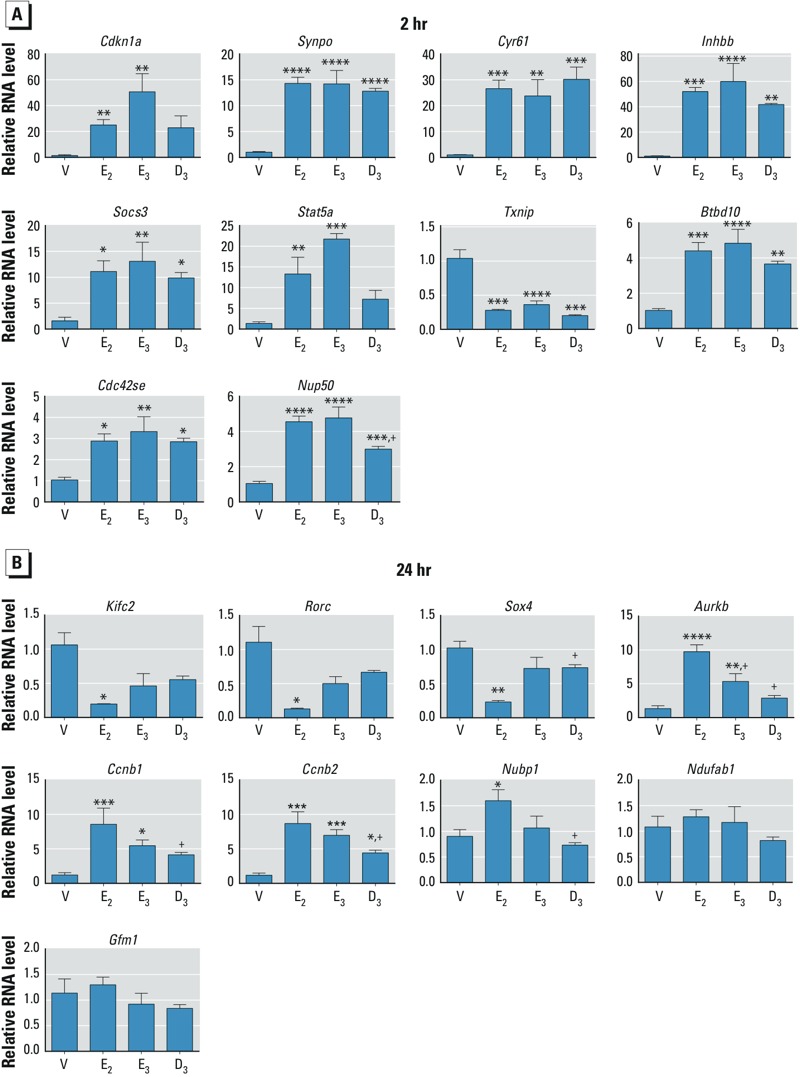
RT-PCR of RNA samples from mice treated for 2 or 24 hr with saline vehicle (V), E_2_, E_3_, or D3 to validate biomarker panels. Values were calculated relative to V and normalized to *Rpl7* (mean ±SE; *n *= 3–6/group). *p < 0.05, **p < 0.01, ***p < 0.001, ****p < 0.0001, and ^+^*p* < 0.05 compared with E_2_ by one-way ANOVA, with multiple comparisons, with Tukey’s multiple test correction, except for *Cdkn1a*, with no multiple test correction (Fisher’s least signicant difference test). (*A*) Transcripts selected from the 2-hr panel to indicate estrogenic response after 2 hr: *Cdkn1a*,**cyclin-dependent kinase inhibitor 1A; *Synpo*, synaptopodin; *Cyr61*, cysteine rich protein 61; *Inhbb*, Inhibin beta-B; *Socs3*, suppressor of cytokine signaling 3, *Stat5a*, signal transducer and activator of transcription 5A; *Txnip*, thioredoxin interacting protein; *Btbd10*, BTB (POZ) domain containing 10; *Cdc42se*, CDC42 small effector 1; *Nup50*, nucleoporin 50. (*B*) Transcripts selected from the 24-hr panel to distinguish long‑ from short-acting estrogens after 24 hr: *Kifc2*, kinesin family member C2; *Rorc*, RAR-related orphan receptor gamma; *Sox4*, SRY-box containing gene 4; *Aurkb, *aurora kinase B; *Ccnb1*, cyclin B1; *Ccnb2*, cyclin B2; *Nubp1*, nucleotide binding protein 1; *Ndufab1*, NADH dehydrogenase (ubiquinone) 1, alpha/beta subcomplex, 1 precursor; *Gfm1*, G elongation factor, mitochondrial 1 precursor.

*RT-PCR and validation of transcript panels*. To validate our potential screening strategy, as shown in Table 1, we evaluated a diarylheptanoid, D3 (Figure 3A) (Suksamrarn et al. 2008), which has traditionally been used by women in Thailand to relieve postmenopausal symptoms. Studies by Winuthayanon et al. (2009, 2013) have suggested that this compound has estrogenic properties. As a test of this concept, we used biological response end points together with probes selected from each biomarker panel. Treatment with D3 resulted in the entry of uterine epithelial cells into S phase, and EdU incorporation and uterine weight was increased to a level comparable with E_2_ after 24 hr (Figure 3B–D). However, the uterine weight after 72 hr was lower than that reached by E_2_ (Figure 3D) and was not statistically significant (*p* = 0.054), as reported in earlier studies (Winuthayanon et al. 2009, 2013). The lower weight increase after 72 hr of treatment was reflected in increased TUNEL-positive cells (Figure 3E,F) as well as in an attenuated LEH increase (Figure 3G). These biological observations are consistent with short-acting estrogenic activity. Therefore, we evaluated transcriptional responses of selected biomarker panel transcripts after 2 or 24 hr of treatment (Figure 4A,B). Of the 10 transcripts from the 2-hr panel we tested, 8 showed similar responses with E_2_, E_3_, or D3, but D3 did not significantly induce two of the transcripts (*Cdkn1a* and *Stat5a*) and induced *Nup50* less robustly than did E_2_ or E_3_ (Figure 4A). In the 24-hr panel, 2 transcripts (*Ndufab1* and *Gfm1*) showed no regulation with any of the tested substances (Figure 4B). The remaining 7 transcripts either showed no response with D3 (*Kifc2*, *Rorc*, *Sox4*, *Ccnb1*, *Nubp1*, and *Aurkb*) or a response that was significant but was blunted compared with the E_2_ response (*Ccnb2*) (Figure 4B). Depending on the transcript, E_3_ exhibited responses similar to those of D3 (*Kifc2*, *Rorc*, and *Sox4*) but in some cases the response was between those of D3 and E_2_ (*Aurkb* and *Ccnb2*).

The RNA samples were then analyzed using a NanoString CodeSet that included 129 biomarkers and 9 housekeeping probes. Most of the probes in each of the panels confirmed the expected patterns (see Supplemental Material, Figure S3), with the 2-hr probes indicating regulation relative to vehicle by E_2_, E_3_ or D3 (see Supplemental Material, Figure S3A) and the 24-hr panel confirming differential regulation by long- versus short-acting estrogens (see Supplemental Material, Figure S3B).

## Discussion

Endocrine disruption describes a property of exogenous chemicals, either natural or man-made, that leads to perturbation of biological function via endogenous endocrine systems. These chemicals have the potential to greatly impact human health and the environment. However, endocrine disruption encompasses broad classes of chemicals that individually affect diverse biological signaling pathways. Thus, there is a need to focus on identifying which substances can affect which molecular events. In the present study, we designed a multipronged approach that combines transcriptional and phenotypic end points in order to evaluate chemicals suspected to affect estrogen signaling. As summarized in Table 1, our study design included treatment of ovariectomized mice with various chemicals and collecting uteri 2 hr or 24 hr after a single injection, or 24 hr after last of three daily injections (72 hr). Substances that are estrogenic increased epithelial cell DNA synthesis at the 24-hr end point and regulated transcripts in the 2-hr panel relative to vehicle or a nonestrogenic chemical at the 2-hr end point. Evaluation of uterine weights at the 24-hr and 72-hr end points could then be used to indicate whether an estrogenic substance is long-acting (weight increases observed) or short-acting (weight increase is attenuated or absent). At the 24-hr end point, regulation of transcripts in the 24-hr panel by long- versus short-acting estrogens was distinct, because short-acting estrogens exhibited impeded regulation of these transcripts. Although the end points utilized in our approach are most relevant to uterine responses, the resulting classification of compounds as short- or long-acting estrogens is important when evaluating potential impacts on other estrogen-sensitive tissues and species. In addition, our approach specifically evaluated ERα-mediated effects; ERα is essential for mouse uterine responses, revealed by the full spectrum of estrogen responsiveness exhibited by ERβ-null mice (Hewitt et al. 2003). Once substances are classified by this approach as short- or long-acting estrogens, subsequent analyses could gauge ERα- and ERβ-mediated activities, as well as responses in other estrogen-sensitive tissues and selective agonist versus antagonist activities. Thus, further analyses subsequent to these screens would be the next steps to evaluate whether xenoestrogens behave as selective estrogen receptor modulators such as tamoxifen (antagonistic in breast cells; agonist activity in endometrial tissue).

A previous study revealed a role for ERα in protecting epithelial cells from undergoing apoptosis subsequent to the initial proliferation response (Winuthayanon et al. 2010). Here, we observed apoptosis of uterine epithelial cells as reflected by ineffective induction of the apoptosis inhibitor *Birc1a*, as well as increased detection of TUNEL-positive cells after 72 hr of treatment with short-acting estrogens (Table 1). This suggests that, similar to what we saw after ablation of epithelial ERα (Winuthayanon et al. 2010), short-acting estrogens are unable to maintain epithelial cell ERα activity following initial proliferation, and thus apoptosis occurs. This selective ability of long-acting estrogens to induce *Birc1a* can initially be seen after 24 hr (data not shown) but is most apparent after 72 hr.

PPT is a known ERα selective agonist but has not previously been classified as short or long acting. In this study, some end points confirmed the estrogenic activity of PPT [DNA synthesis (Figure 2A) and 2-hr transcript regulation (see Supplemental Material, Figure S2A)]. However, on the basis of end points, it was difficult to pin down the activity as short or long acting. Uterine weight did not increase after 24 hr, but a significant uterine weight increase occurred after 72 hr; however, this increase was attenuated compared with that of long-acting estrogens (Figure 2D). In addition, PPT was significantly less effective at inducing *Ltf* (Figure 2G), but it induced the *Birc1a* apoptosis inhibitor equally as well as long-acting estrogens (Figure 2F), which likely explains the observed weight increase after 72 hr. *Birc1a* was not significantly increased by PPT after 24 hr (data not shown), and PPT-treated uteri exhibited TUNEL-positive cells at a level similar to that of E_3_ (Figure 2H,I). At 24 hr PPT increased *Ltf* less effectively than E_2_ or DES (not shown). On all the basis of the observed end points, we classify PPT as an intermediate compound that is not a long- or short-acting estrogen. We anticipate that other compounds tested in future analyses could also exhibit intermediate characteristics, and this will be vital in predicting the risks of harmful effects, because less severe effects would be predicted for a shorter-acting estrogen, but one with intermediate characteristics might need closer consideration.

Our use of a diarylheptanoid (D3) compound isolated from a traditional Thai medicinal plant validated the approach outlined in Table 1. Most, but not all, transcripts from the biomarker panel probes tested by RT-PCR produced the expected transcript regulation patterns with E_3_ or D3. A more comprehensive analysis using the NanoString code set indicated that our biomarker panel shows promise for distinguishing estrogenicity and relative strength of endocrine disruptors. The regulation of three of the transcripts selected from the 24-hr panel (*Nubp1*, *Ndufab1*, *Gfm1*) could not be confirmed by RT-PCR. In the NanoString analysis, *Nubp1* showed slight induction by E_2_, *Ndufab1* was minimally regulated, and E_2_ selective induction of *Gfm1* regulation was readily apparent (see Supplemental Material, Supplemental XLSX, Table S5). Differences may reflect disparities between the probes used on the microarray platform, NanoString probes, and the primer sets employed for RT-PCR.

Other approaches have focused on high throughput screens to identify potential endocrine disruptors, such as the ambitious Tox21 project at the National Toxicology Program. Tox21 aims to evaluate toxicants using robotic screens based on *in vitro* end points (Huang et al. 2011). Initial efforts include focus on nuclear receptor interactions using ligand binding domains (LBD) of 10 different nuclear receptors in a Gal4-based response screen of a library containing approximately 3,000 environmentally relevant chemicals (Huang et al. 2011). The objective of the Tox21 approach is a broad, high throughput initial evaluation to identify chemicals for further consideration. The approach we have outlined here would be a logical next step for additional evaluation of chemicals that are positive in the Tox21 nuclear receptor assay and interact with ERα. The primary information obtained from the initial Tox21 screen reflects the chemical’s ability to bind to and modulate the activity of the LBD of the ERα (the assay included only this portion of the receptor) and measures regulation of exogenous reporter gene activity. Such an approach lacks the inter- and intramolecular interactions mediated in the context of the full-length ERα molecule and representation of *in vivo* responses of endogenous genes. In addition, the Tox21 assay will not capture subtleties of estrogenic response, such as those typified by long- versus short-acting estrogenic activities. Our assay scheme is useful in the context of a “second step” after an initial broad screen because the Tox21 approach is more realistic for evaluating thousands of chemicals. Our approach focuses on end points observed in mouse uterine tissue and thus may not reflect effects in other estrogen target tissues, such as breast or liver, or effects in other species. However, our panel of phenotypic and transcription response is an appropriate partner to the broad Tox 21 approach and could be used to fine tune and finesse the mechanistic particulars and biological effects in a real whole animal context.

Moggs et al. (2004) combined bioinformatics and phenotypic anchoring approaches to a similar model, the immature mouse uterus. Although their approach is very useful toward defining transcriptional profiles that underlie phenotypic observations, it is not designed for the type of evaluation of mechanisms of action we focused on in our study. Watanabe et al. (2004) also used the ovariectomized mouse model to compare transcriptional responses of uterine tissue 6 hr after injection of E_2_ or 2,3,7,8-tetrachlorodibenzo-*p*-dioxin (TCDD). In their study, some uterine transcripts were regulated in a similar manner by E_2_ or TCDD, suggesting estrogenic activity of TCDD. Many of the estrogen-responsive transcripts showed a weaker response with TCDD. Unlike our approach, however, their screen was not a comprehensive attempt to select probes for screening. In another study Hong et al. (2006) used the immature rat model to evaluate uterine transcript profiles 24 hr after the last of three daily injections of E_2_, DES, or the endocrine disruptors BPA, genistein, and octylphenol or nonylphenol. Unlike the present study, the transcript profile for each substance was distinct, with few commonly regulated probes. This is especially surprising in the case of DES- versus E_2_-regulated transcripts, which revealed only 126 common probes out of 555 E_2_-regulated and 674 DES-regulated probes (Hong et al. 2006), whereas we observed similar uterine responses to 72-hr E_2_ or DES in our model (Figure 1). RT-PCR analysis of several transcripts revealed responses consistent with our observations, with E_2_ and DES inducing transcripts at the 72-hr time point and BPA showing no induction (Hong et al. 2006). Naciff et al. (2002) developed a gestational exposure model to evaluate uterine transcripts in gestational day (GD) 20 rat pups 2 hr after the last daily injection of ethynyl E_2_, BPA, or genistein on GDs 11–20. The approach of Naciff et al. is complementary to ours: They sought to develop a transcriptional profile “fingerprint” as evidence of gestational exposures and we focused on discerning potential modes of action reflecting biochemical characteristics.

*In vitro* models have also been developed utilizing Ishikawa cells, a human endometrial cancer cell line. Boehme et al. (2009) compared transcriptional profiles of ERα-positive and -negative Ishikawa cells dosed for 24 hr with the estrogenic chemicals DES, BPA, genistein, zearalenone, resveratrol, or *o,p*´-dichlorodiphenyltrichlorethane (DDT), or with the ER antagonist ICI 182,780. From their analysis, they derived a panel of estrogen-responsive transcripts as candidates for screening of estrogenic activity. Similar to our observations, BPA showed weaker activity (Boehme et al. 2009). Naciff et al. (2010) compared transcriptional profiles of ERα-positive Ishikawa cells treated with ethynyl E_2_ or BPA for 8 hr, 24 hr, or 48 hr, with the focus on developing a relevant *in vitro* estrogen response model. These authors identified 307 commonly regulated probes as candidate biomarkers of potential estrogenic activity. They further compared their BPA-responsive Ishikawa transcripts with uterine transcriptional profiles from juvenile rats dosed with estrogen for 8, 24, or 48 hr and observed 362 commonly regulated transcripts (Naciff et al. 2010). Using another approach, Bredhult et al. (2009) reported transcriptional analysis of primary cultures of human endometrial endothelial cells (HEEC) 24 hr after treatment with BPA, which led to decreased HEEC growth. They did not observe effects on known estrogen-responsive transcripts; however, because HEECs are ERα negative and express ERβ, responses might reflect, in part, ERβ targets. Abot et al. (2013) derived a panel of 40 estrogen-responsive transcripts from published mouse uterus microarray data sets and also included evaluation of phenotypic end points, including uterine weights and the proliferative marker Ki67. Their study was focused on the role of the AF1 domain of ERα in the uterus, which has been shown to be an AF1-stimulated tissue (Arao et al. 2011). Abot et al. (2013) were able to use tamoxifen (an AF1-dependent estrogenic ligand) and mutations that disabled AF1 activity of the ERα to demonstrate roles of AF1 as illustrated by changes in transcription or biological end points. However, these useful studies are not designed to further screen mechanisms underlying activity or potential biological outcomes.

## Conclusion

The approach presented here (outlined in Table 1) could be used to evaluate potentially estrogenic chemicals and could indicate possible biological mechanisms that would improve our understanding of the potential impacts on human health.

## Supplemental Material

(9.1 MB) PDF

(48 KB) PDF

## References

[r1] Abot A, Fontaine C, Raymond-Letron I, Flouriot G, Adlanmerini M, Buscato M (2013). The AF-1 activation function of estrogen receptor α is necessary and sufficient for uterine epithelial cell proliferation in vivo.. Endocrinology.

[r2] Arao Y, Hamilton KJ, Ray MK, Scott G, Mishina Y, Korach KS (2011). Estrogen receptor α AF-2 mutation results in antagonist reversal and reveals tissue selective function of estrogen receptor modulators.. Proc Natl Acad Sci USA.

[r3] Boehme K, Simon S, Mueller SO (2009). Gene expression profiling in Ishikawa cells: a fingerprint for estrogen active compounds.. Toxicol Appl Pharmacol.

[r4] Bredhult C, Sahlin L, Olovsson M (2009). Gene expression analysis of human endometrial endothelial cells exposed to bisphenol A.. Reprod Toxicol.

[r5] Chou JW Zhou T Kaufmann WK Paules RS Bushel PR 2007 Extracting gene expression patterns and identifying co-expressed genes from microarray data reveals biologically responsive processes. BMC Bioinformatics 8 427; 10.1186/1471-2105-8-427 17980031 PMC2194742

[r6] Clark JH, Markaverich BM (1984). The agonistic and antagonistic actions of estriol.. J Steroid Biochem.

[r7] Gormley M Dampier W Ertel A Karacali B Tozeren A 2007 Prediction potential of candidate biomarker sets identified and validated on gene expression data from multiple datasets. BMC Bioinformatics 8 415; 10.1186/1471-2105-8-415 17963508 PMC2211325

[r8] Hewitt SC, Deroo BJ, Hansen K, Collins J, Grissom S, Afshari CA (2003). Estrogen receptor-dependent genomic responses in the uterus mirror the biphasic physiological response to estrogen.. Mol Endocrinol.

[r9] Hewitt SC, Kissling GE, Fieselman KE, Jayes FL, Gerrish KE, Korach KS (2010). Biological and biochemical consequences of global deletion of exon 3 from the ERα gene.. FASEB J.

[r10] Hewitt SC Korach KS 2011 Estrogenic activity of bisphenol A and 2,2-bis(*p*-hydroxyphenyl)-1,1,1-trichloroethane (HPTE) demonstrated in mouse uterine gene profiles. Environ Health Perspect 119 63 70; 10.1289/ehp.1002347 20826375 PMC3018502

[r11] Hewitt SC, Li L, Grimm SA, Chen Y, Liu L, Li Y (2012). Research resource: whole-genome estrogen receptor α binding in mouse uterine tissue revealed by ChIP-seq.. Mol Endocrinol.

[r12] Hewitt SC, O’Brien JE, Jameson JL, Kissling GE, Korach KS (2009). Selective disruption of ERα DNA-binding activity alters uterine responsiveness to estradiol.. Mol Endocrinol.

[r13] Hong EJ Park SH Choi KC Leung PC Jeung EB 2006 Identification of estrogen-regulated genes by microarray analysis of the uterus of immature rats exposed to endocrine disrupting chemicals. Reprod Biol Endocrinol 4 49; 10.1186/1477-7827-4-49 17010207 PMC1594574

[r14] Huang R Xia M Cho MH Sakamuru S Shinn P Houck KA 2011 Chemical genomics profiling of environmental chemical modulation of human nuclear receptors. Environ Health Perspect 119 1142 1148; 10.1289/ehp.1002952 21543282 PMC3237348

[r15] Katzenellenbogen BS (1984). Biology and receptor interactions of estriol and estriol derivatives *in vitro* and *in vivo*.. J Steroid Biochem.

[r16] Katzenellenbogen BS, Bhakoo HS, Ferguson ER, Lan NC, Tatee T, Tsai TS (1979). Estrogen and antiestrogen action in reproductive tissues and tumors.. Recent Prog Horm Res.

[r17] Klotz DM, Hewitt SC, Korach KS, Diaugustine RP (2000). Activation of a uterine insulin-like growth factor I signaling pathway by clinical and environmental estrogens: requirement of estrogen receptor-α.. Endocrinology.

[r18] Moggs JG Tinwell H Spurway T Chang HS Pate I Lim FL 2004 Phenotypic anchoring of gene expression changes during estrogen-induced uterine growth. Environ Health Perspect 112 1589 1606; 10.1289/txg.7345 15598610 PMC1247656

[r19] Naciff JM, Jump ML, Torontalli SM, Carr GJ, Tiesman JP, Overmann GJ (2002). Gene expression profile induced by 17α-ethynyl estradiol, bisphenol A, and genistein in the developing female reproductive system of the rat.. Toxicol Sci.

[r20] Naciff JM, Khambatta ZS, Reichling TD, Carr GJ, Tiesman JP, Singleton DW (2010). The genomic response of Ishikawa cells to bisphenol A exposure is dose- and time-dependent.. Toxicology.

[r21] National Research Council. (2011). Guide for the Care and Use of Laboratory Animals. 8th ed. Washington, DC:National Academies Press.. http://www.nap.edu/openbook.php?record_id=12910.

[r22] Safe SH, Pallaroni L, Yoon K, Gaido K, Ross S, Saville B (2001). Toxicology of environmental estrogens.. Reprod Fertil Dev.

[r23] Sinkevicius KW, Burdette JE, Woloszyn K, Hewitt SC, Hamilton K, Sugg SL (2008). An estrogen receptor-α knock-in mutation provides evidence of ligand-independent signaling and allows modulation of ligand-induced pathways *in vivo*.. Endocrinology.

[r24] Suksamrarn A, Ponglikitmongkol M, Wongkrajang K, Chindaduang A, Kittidanairak S, Jankam A (2008). Diarylheptanoids, new phytoestrogens from the rhizomes of *Curcuma comosa*: isolation, chemical modification and estrogenic activity evaluation.. Bioorg Med Chem.

[r25] Watanabe H, Suzuki A, Goto M, Ohsako S, Tohyama C, Handa H (2004). Comparative uterine gene expression analysis after dioxin and estradiol administration.. J Mol Endocrinol.

[r26] Winuthayanon W, Hewitt SC, Orvis GD, Behringer RR, Korach KS (2010). Uterine epithelial estrogen receptor α is dispensable for proliferation but essential for complete biological and biochemical responses.. Proc Natl Acad Sci USA.

[r27] Winuthayanon W Piyachaturawat P Suksamrarn A Burns KA Arao Y Hewitt SC 2013 The natural estrogenic compound diarylheptanoid (D3): *in vitro* mechanisms of action and *in vivo* uterine responses via estrogen receptor α. Environ Health Perspect 121 433 439; 10.1289/ehp.1206122 23552522 PMC3620745

[r28] Winuthayanon W Piyachaturawat P Suksamrarn A Ponglikitmongkol M Arao Y Hewitt SC 2009 Diarylheptanoid phytoestrogens isolated from the medicinal plant *Curcuma comosa*: biologic actions *in vitro* and *in vivo* indicate estrogen receptor-dependent mechanisms. Environ Health Perspect 117 1155 1161; 10.1289/ehp.0900613 19654927 PMC2717144

