# Supplementary material for: Development of Phenotypic and Transcriptional Biomarkers to Evaluate Relative Activity of Potentially Estrogenic Chemicals in Ovariectomized Mice
Source: Environ Health Perspect. 2015 Jan 9;123(4):344–52. doi: 10.1289/ehp.1307935 (PMC4383572; doi:10.1289/ehp.1307935)
Supplement: (9.1 MB) PDF [file ehp.1307935.s001.508.pdf]

Supplemental Material

Development of Phenotypic and Transcriptional Biomarkers to Evaluate Relative Activity of Potentially Estrogenic Chemicals in Ovariectomized Mice

Sylvia C. Hewitt, Wipawee Winuthayanon, Brianna Pockette, Robnet T. Kerns, Julie F. Foley, Norris Flagler, Elizabeth Ney, Apichart Suksamrarn, Pawinee Piyachaturawat, Pierre R. Bushel, and Kenneth S. Korach

Methods

EPIG Analysis

An analysis of variance (ANOVA) model was applied to remove batch effects by project number, while retaining treatment, time and (treatment) x (time) effects. Principal Component Analysis (PCA) output following this batch correction is shown in Figure S4. All the control saline treated samples were collected after 24 h. The batch corrected datasets were analyzed using the Extracting Patterns and Identifying co-expressed Genes (EPIG) tool (Chou et al. 2007) resulting in 16 patterns (Figure 1 A-B). Briefly, for each probe, an ANOVA mixed effects linear model, $Y_{ijkl} = \mu + \alpha_i + \beta_j + \gamma_l + \alpha\beta_{ij} + \varepsilon_{ijkl}$, where μ is the grand mean expression and Y_{ijkl} represents the quantile normalized and log2 transformed pixel intensity measurement of the ith treatment, jth time, kth (project random effect) and lth sample, was applied to batch correct the data. ε_{ijkl} represents the random error. The errors are assumed to be normally and independently distributed, with a mean of 0 and a standard deviation of δ for all measurements. Using the quantile normalized, log2 transformed and batch corrected data, the vehicle control group was set as the reference for each treatment. The average of the data from the replicates of the vehicle controls were aligned to zero as a baseline, with the treated samples adjusted by the same amount. EPIG evaluates each probe's expression profile for categorization into a pattern based on the following parameters: correlation (r), signal magnitude (mag) and signal-to-noise ratio (SNR). A Mag = 0.5 is equivalent to a 1.5 fold change; an $r > 0.8$ is reasonably good correlation and a SNR ≥ 3 is considered to be a robust response. Note that a SNR = 3 equates to a *p*-value = 3.72E-09. To identify co-expressed genes within patterns that exhibit a response to treatments, EPIG filtered each gene expression profile based on the following criteria: correlation $r \geq 0.8$

and [(mag ≥ 0.5 and SNR ≥ 3) or (t > 4.21)]. t is a threshold set equal to (mag)*(-log10 SNR *p*-value).

Deriving biomarker panels

To identify transcripts probes as biomarkers we used the above ANOVA model and transformed data from the 21 estrogenic samples and 14 vehicle control samples (Table S1). Contrasts were performed to statistically compare the difference between the means of the 2 h estrogenic response and control expression measurements. A Bonferoni –corrected $p<0.01$ cutoff to account for multiple corrections yielded 12038 probes. PCA of the samples using these 12038 probes revealed that PC1 accounted for 76.8% of the variance, compared to 35.2% for the entire 41174 probe chip (Table S2). The 25 probes with the highest and lowest loading within PC1 were selected from these 12038. To validate, a slightly different PCA implementation was used and bootstrap resampling to test variance of the PC1 for the 50 probe list versus a random list of 50 probes from the entire chip. This validation test of 50,000 iterations resulted in a *p*-value <2E-.05. A second validation involved hierarchical clustering of the expression of the 50 probes for all 35 samples (Z-scores: expression data standardized to mean = 0 and SD = 1; Figure S2A), resulting in two distinct clusters separating vehicle-treated samples from estrogen-like-treated samples. To test the significance of the clustering, 300,000 random selections of 50 probes from the 41174 microarray chip for k-means clustering into two partitions were performed yielding a *p*-value of 3.3E-6 reflecting the chance (probability) that the clustering of the samples would be as stable (Famili et al. 2004) as the 50 probes estrogenic response biomarker panel.

To create a panel of 50 probes that distinguish long-acting from short-acting estrogens based on expression at 24 h, the 58 chips listed in Table S3 were used. The ANOVA model incorporating the normalized, transformed and corrected data from all 58 chips was used to contrast long-

acting vs. short-acting responses at 24 h, also accounting for time and type (substance) factors. A Bonferoni corrected *p*-value < 0.01 cutoff yielded 9401 probes (Table S4). The 25 probes with the highest and lowest loadings within PC1 were selected. To validate, a slightly different PCA implementation was used and bootstrap resampling to test variance of the first PC for the 50 probe list versus a random list of 50 probes from the entire chip. This validation test of 10000 iterations gave *p*-value<1E-05. A second validation involved hierarchical clustering of the expression of the 50 probes for 24 h samples (Z-scores: expression data standardized to mean = 0 and SD=1). For this validation, PPT 24 h samples were not included, as this compound is classified as an ERα selective agonist, but has not yet been classified as long- or short-acting. BPA and HPTE, which we previously demonstrated to be short-acting estrogens (Hewitt and Korach 2011), were included. The cluster of 24 h samples only (Figure S2B), revealed two distinct clusters separating short-acting estrogen-like samples from long-acting estrogen-like samples. To test the significance of the clustering, 1000000 selections of 50 probes from the 41174 probe microarray chip for k-means clustering into two partitions were performed yielding a *p*-value of 1E-6 reflecting the chance (probability) that the clustering of the samples would be as stable (Famili et al. 2004) as the 50 probes strength of estrogenic response biomarker panel.

Figure S1. Doses used.

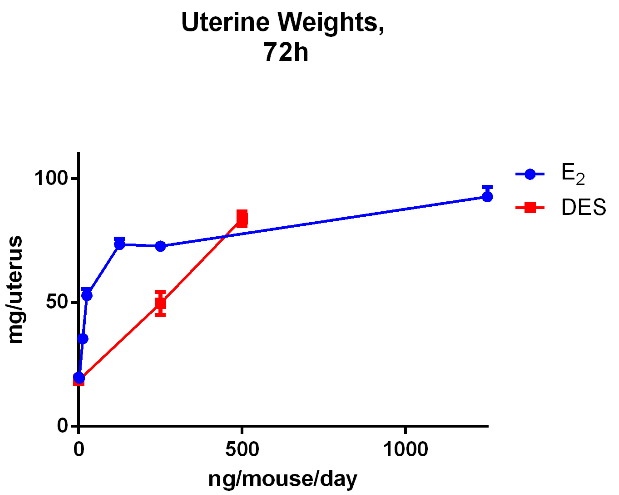

Uterine weights after dosing for 72 h with E_2 or DES. 250 ng/mouse/day was selected for studies.

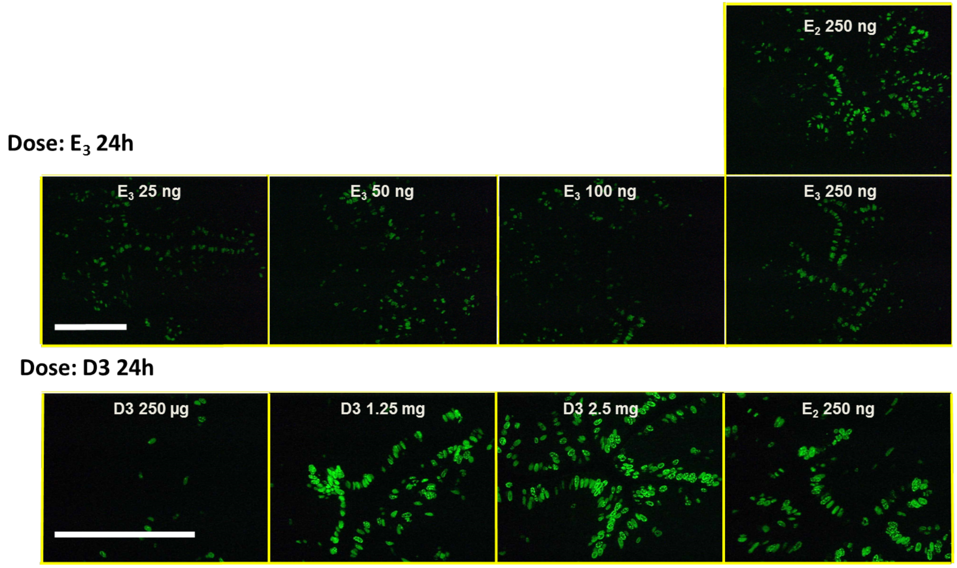

EdU incorporation induced after 24 h treatments with E_3 or D3, compared to 250 ng of E_2. Bar = 0.1 mM

Figure S2

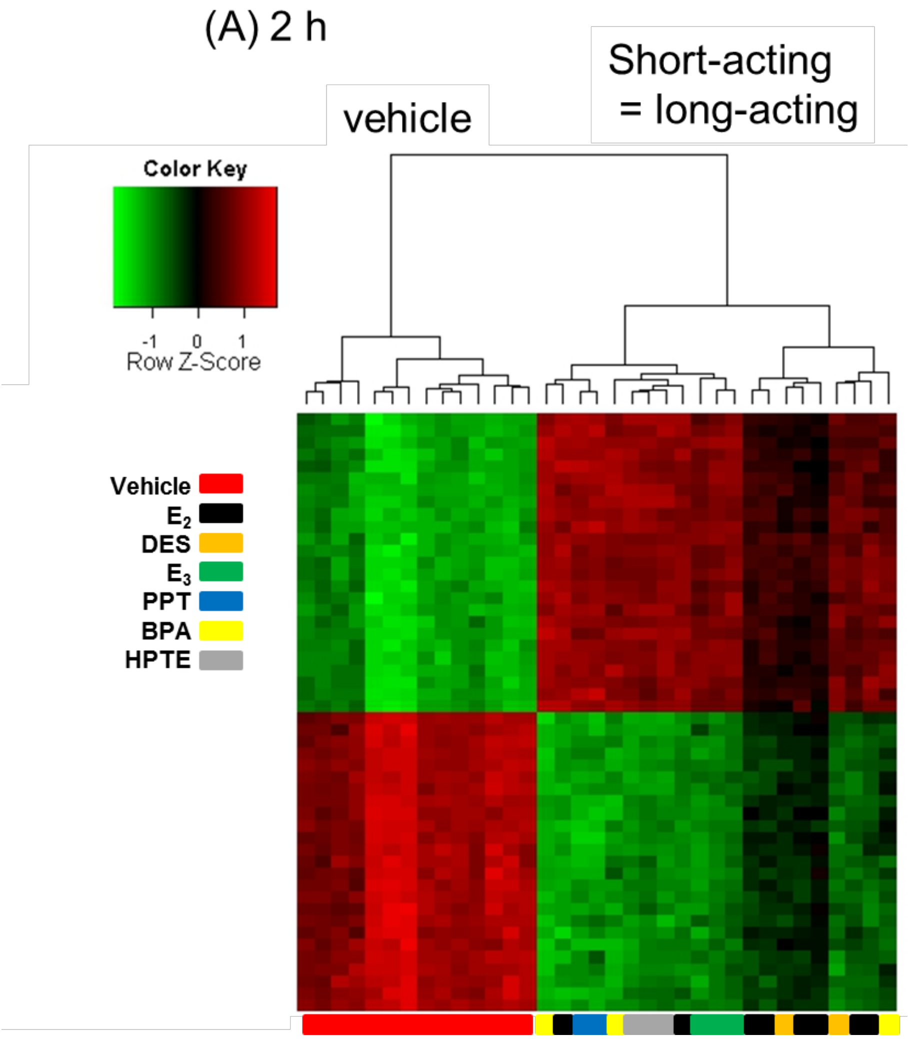
(A) 2 h
vehicle
Short-acting
= long-acting
Color Key
-1 0 1
Row Z-Score
Vehicle
E2
DES
E3
PPT
BPA
HPTE

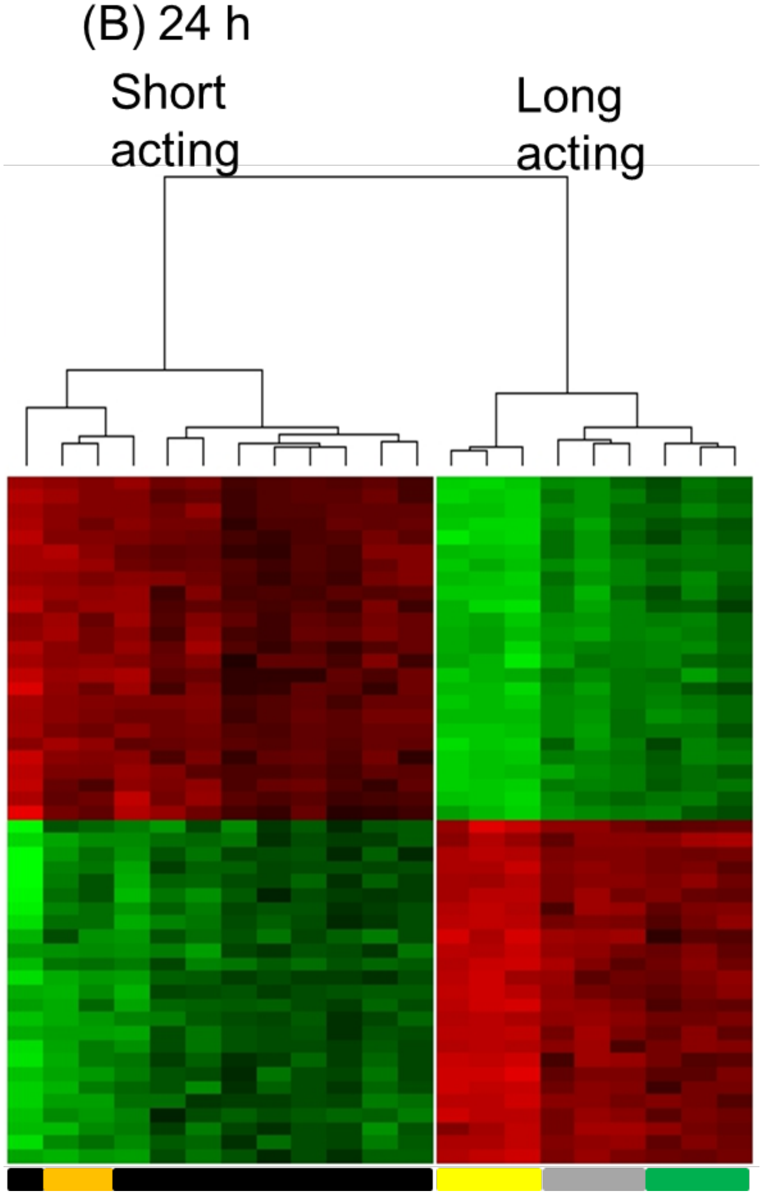

Figure S2. Clusters of biomarker panel probes using microarray datasets. From datasets deposited in GEO; GSE24525 and GSE23241 (Hewitt and Korach 2011), GSE23072 (Hewitt et al. 2010), and GSE61921 not previously published. **A.** Hierarchical cluster of 50 probes differentially expressed after 2 h relative to V with both long- and short-acting estrogens V (saline, red, 14 samples) E_2 (black, 8 samples), E_3 (green, 3 samples), PPT (blue, 2 samples), DES (orange, 2 samples), BPA (yellow, 3 samples), or HPTE (grey, 3 samples). Probes are listed in Table S6. **B.** Hierarchical cluster of 50 probes that distinguish long- from short-acting estrogens after 24 h. E_2 (10 samples), E_3 (green, 3 samples), DES orange, 2 samples), BPA (yellow, 3 samples), or HPTE (grey, 3 samples). Probes are listed in Table S7.

Figure S3

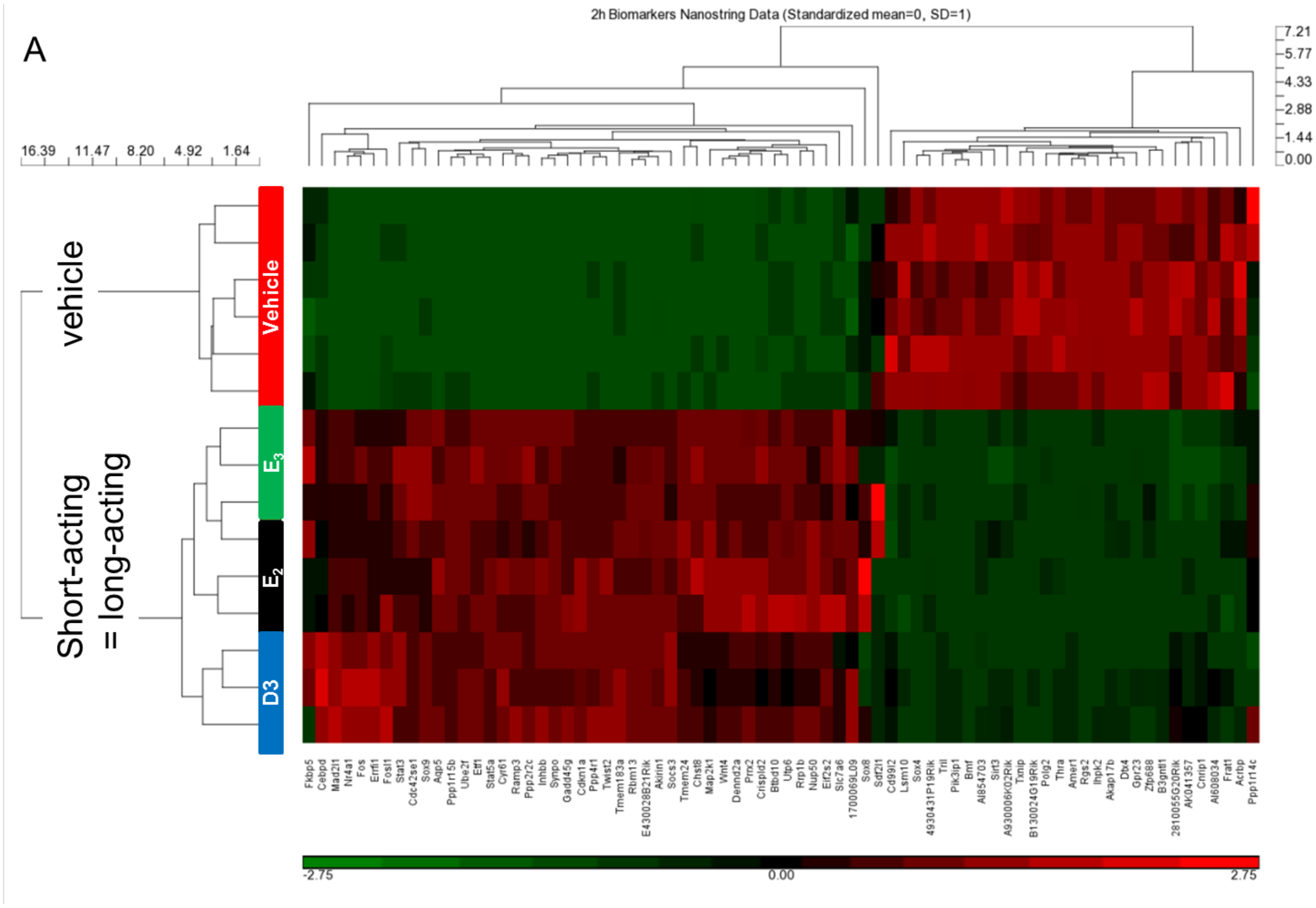
A
2h Biomarkers Nanostring Data (Standardized mean=0, SD=1)
7.21
5.77
4.33
2.88
1.44
0.00
16.39
11.47
8.20
4.92
1.64
vehicle
Short-acting
= long-acting
Vehicle
E_3
E_2
D3
Fkbp5
Cebpd
Mad2l1
Nr4a1
Fos
Errfi1
Fosl1
Stat3
Cdc42se1
Sox9
Aqp5
Ppp1r15b
Ube2f
Etf1
Stat5a
Cyr61
Ramp3
Ppp2r2c
Inhbb
Synpo
Gadd45g
Cdkn1a
Ppp4r1
Twist2
Tmem183a
Rbm13
E430028B21Rik
Akirin1
Socs3
Tmem24
Chst8
Map2k1
Wnt4
Dennd2a
Prrx2
Crispld2
Btbd10
Utp6
Rrp1b
Nup50
Eif2s2
Slc7a6
1700069L09
Sox8
Sdf2l1
Cd99l2
Lsm10
Sox4
4930431P19Rik
Tril
Pik3ip1
Bmf
AI854703
Sirt3
A930006K02Rik
Txnip
B130024G19Rik
Polg2
Thra
Amer1
Rgs2
Ihpk2
Akap17b
Dtx4
Gpr23
Zfp688
B3gntl1
2810055G20Rik
AK041357
Cnrip1
AI608034
Frat1
Acrbp
Ppp1r14c
-2.75
0.00
2.75

B

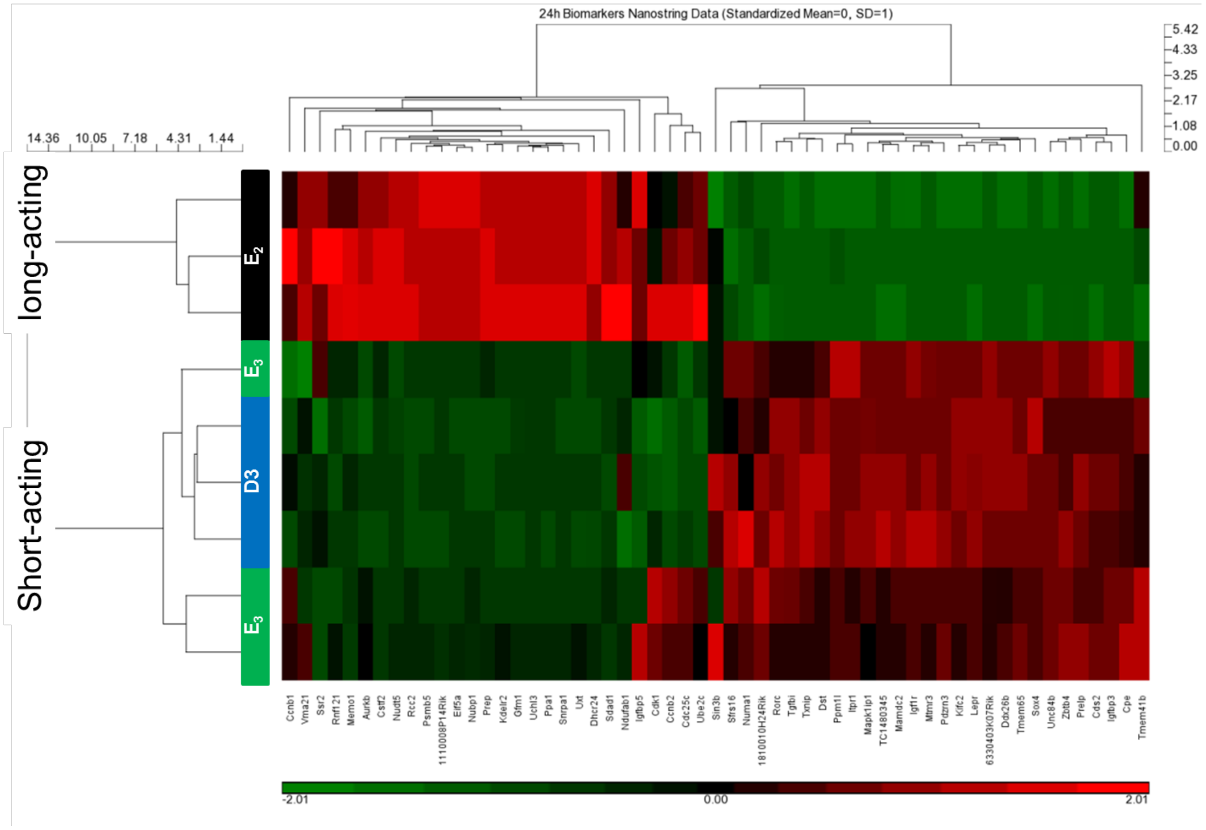

Figure S3. Nanostring evaluation of biomarker panels. Standardized output from analysis of 2 h **(A)** and 24 h **(B)** biomarker panels demonstrating validity of probes to distinguish estrogens (A) and short- vs. long-acting estrogens (B). Normalized Nanostring data were filtered for 2 h or 24 h biomarker probes and hierarchical clusters were constructed with values standardized for each probe to mean=0 and SD=1. (A). Vehicle (saline, red; n=6) and 2 h E_2 (black), E_3 (green), or D3 (blue) samples (each n=3). (B). 24 h E_2 (black), E_3 (green), or D3 (blue) samples (each n=3).

Figure S4

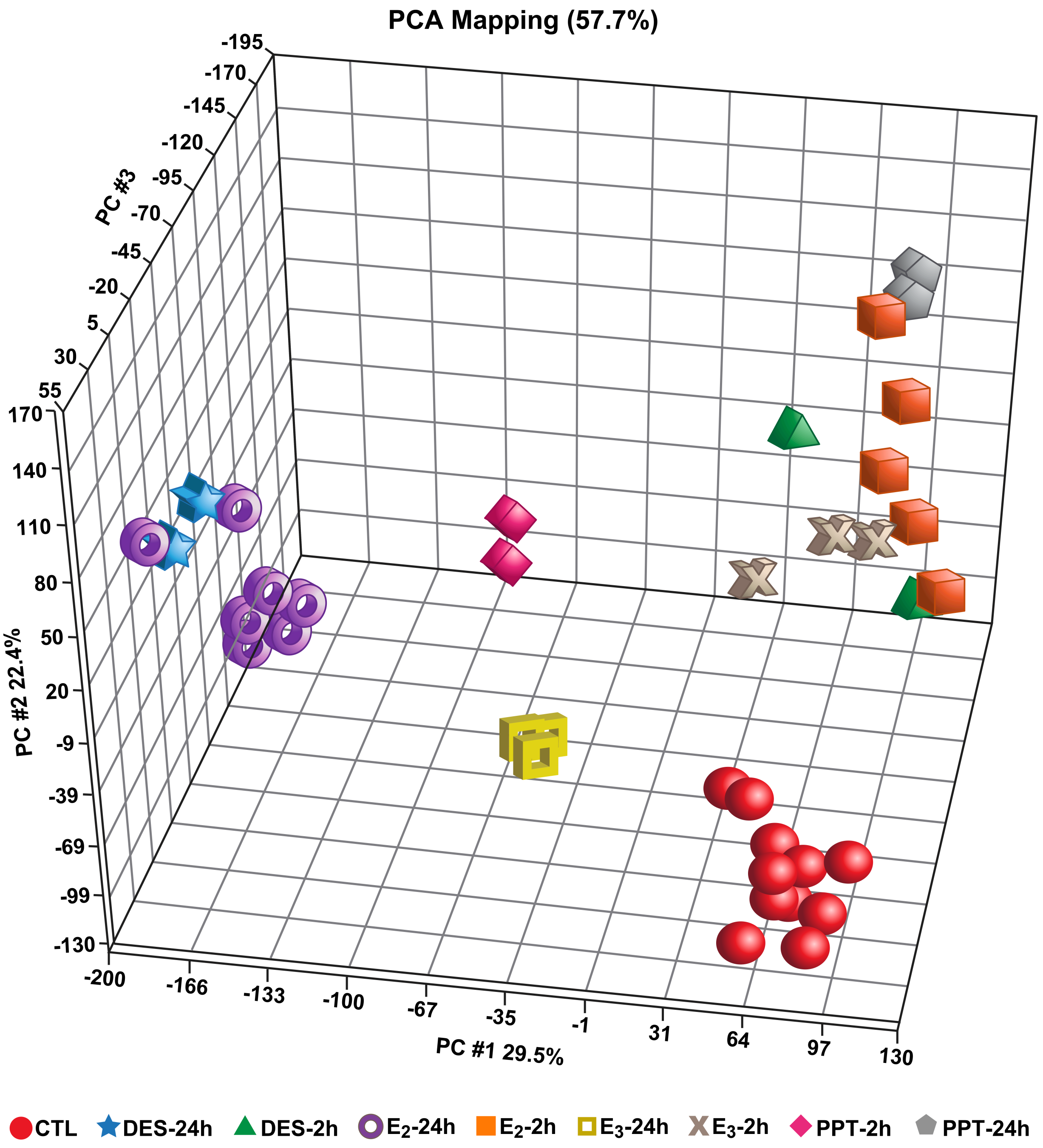

Figure S4. PCA plot of batch corrected samples for EPIG analysis. As described in Methods.

Table S1. Combined analysis: estrogenic 2h.

Treatment	Time (hours)	Experiment name	Estrogen class	*n*
GEO GSE24525 sample descriptions (n=12) (scanned 8/19/2010; 8/25/2010)				
Estradiol	2	E2_2h	Long-Acting	3
Bisphenol A	2	BPA_2h	Short-Acting	3
2,2-bis(*p*-hydroxyphenyl)-1,1,1-trichloroethane	2	HPTE_2h	Short-Acting	3
Vehicle	24	CTL	N/A	3
GEO GSE23072 sample descriptions (n=6) (scanned 10/9/2008)				
Estradiol	2	E2_2h	Long-Acting	3
Vehicle (saline)	24	CTL	N/A	3
GEO GSE61921 sample descriptions (n=8) (scanned 5/14/2010)				
Estradiol	2	E2_2h	Long-Acting	2
Diethylstilbesterol	2	DES_2h	Long-Acting	2
Propyl Pyrazole Triol	2	PPT_2h	not classified	2
Vehicle (saline)	24	CTL	none	2
GEO GSE23241 sample descriptions (n=9) (scanned 12/8/2009)				
Estriol	2	E3_2h	Short-Acting	3
Saline Vehicle	24	CTL	none	3
Saline + DMSO	24	CTL	none	3

GSE24525 and GSE23241 published in (Hewitt and Korach 2011). GSE23072 published in (Hewitt et al. 2010). GSE61921 not previously published.

Table S2. 24h Combined analysis: long-acting vs. short-acting estrogens.

Treatment	Time (hours)	Experiment name	Estrogen class	*n*
GEO GSE24525 sample descriptions (n=20) (scanned 8/19/2010; 8/25/2010)				
Estradiol	2	E2_2h	Long-Acting	3
Estradiol	24	E2_24h	Long-Acting	2
Bisphenol A	2	BPA_2h	Short-Acting	3
Bisphenol A	24	BPA_24h	Short-Acting	3
2,2-bis(*p*-hydroxyphenyl)-1,1,1-trichloroethane	2	HPTE_2h	Short-Acting	3
2,2-bis(*p*-hydroxyphenyl)-1,1,1-trichloroethane	24	HPTE_24h	Short-Acting	3
Vehicle (saline)	24	CTL	N/A	3
GEO GSE23072 sample descriptions (n=9) (scanned 10/9/2008)				
Estradiol	2	E2_2h	Long-Acting	3
Estradiol	24	E2_24h	Long-Acting	3
Vehicle (saline)	24	CTL	N/A	3
GEO GSE61921sample descriptions (n=14) (scanned 5/14/2010)				
Estradiol	2	E2_2h	Long-Acting	2
Estradiol	24	E2_24h	Long-Acting	2
Diethylstilbesterol	2	DES_2h	Long-Acting	2
Diethylstilbesterol	24	DES_24h	Long-Acting	2
Propyl Pyrazole Triol	2	PPT_2h	not classified	2
Propyl Pyrazole Triol	24	PPT_24h	not classified	2
Vehicle (saline)	24	CTL	N/A	2
GEO GSE23241 sample descriptions (n=15) (scanned 12/8/2009)				
Estradiol	24	E2_24h	Long-Acting	3
Estriol	2	E3_2h	Short-Acting	3
Estriol	24	E3_24h	Short-Acting	3
Saline Vehicle	24	CTL	none	3
Saline + DMSO	24	CTL	none	3

GSE24525 and GSE23241 published in (Hewitt and Korach 2011). GSE23072 published in (Hewitt et al. 2010). GSE61921 not previously published.

Table S3. Summary of analysis used to design 2 h Bioset.

Description	Probes	P1 (%)
Start	41174	35.2
PCA ANOVA	12038	76.8
Top 50 loadings	50	97.9

Table S4. Summary of analysis used to design 24 h Bioset.

Description	Probes	2D PCA (%)
Start	41174	48.4
Random sample	50	43-47
PCA ANOVA	9401	80.2
Top 50 loadings	50	96.7

References

Chou JW, Zhou T, Kaufmann WK, Paules RS, Bushel PR. 2007. Extracting gene expression patterns and identifying co-expressed genes from microarray data reveals biologically responsive processes. BMC Bioinformatics 8:427.

Famili AF, Liu G, Liu Z. 2004. Evaluation and optimization of clustering in gene expression data analysis. Bioinformatics 20:1535-1545.

Hewitt SC, Kissling GE, Fieselman KE, Jayes FL, Gerrish KE, Korach KS. 2010. Biological and biochemical consequences of global deletion of exon 3 from the er alpha gene. Faseb J 24:4660-4667.

Hewitt SC, Korach KS. 2011. Estrogenic activity of bisphenol a and 2,2-bis(p-hydroxyphenyl)-1,1,1-trichloroethane (hpte) demonstrated in mouse uterine gene profiles. Environ Health Perspect 119:63-70.
